# Supplementary material for: A Correlation between 3′-UTR of OXA1 Gene and Yeast Mitochondrial Translation
Source: J Fungi (Basel). 2023 Apr 5;9(4):445. doi: 10.3390/jof9040445 (PMC10143089; doi:10.3390/jof9040445)
Supplement: Supplementary file 1 [file jof-09-00445-s001.zip › jof-2269867-supplementary.pdf]

Table S1. Exceptions to the universal genetic code in yeast mitochondria

Codon	Standard Amino Acid	Mitochondrial Exception Amino Acid
AUA	Isoleucine	Methionine
UGA	Stop codon	Tryptophan
CUA, CUC, CUG, CUU	Leucine	Threonine
